# Lung Involvement in Primary Sjögren's Syndrome—An Under-Diagnosed Entity

**DOI:** 10.3389/fmed.2020.00332

**Published:** 2020-07-16

**Authors:** Georgios Sogkas, Stefanie Hirsch, Karen Maria Olsson, Jan B. Hinrichs, Thea Thiele, Tabea Seeliger, Thomas Skripuletz, Reinhold Ernst Schmidt, Torsten Witte, Alexandra Jablonka, Diana Ernst

**Affiliations:** ^1^Department of Immunology and Rheumatology, Medical School Hannover, Hanover, Germany; ^2^Department of Respiratory Medicine, Hannover Medical School, Hanover, Germany; ^3^BREATH German Centre for Lung Research (DZL), Hanover, Germany; ^4^Department of Diagnostic and Interventional Radiology, Hannover Medical School, Hanover, Germany; ^5^Department of Neurology, Hannover Medical School, Hanover, Germany

**Keywords:** interstitial lung disease (ILD), lung fibrosis, sicca syndrome, ESSDAI**—**EULAR Sjögren's Syndrome Disease Activity Index, Sjögren's Syndrome (SS)

## Abstract

Interstitial lung disease (ILD) represents a frequent extra-glandular manifestation of primary Sjögren's Syndrome (pSS). Limited published data regarding phenotyping and treatment exists. Advances in managing specific ILD phenotypes have not been comprehensively explored in patients with coexisting pSS. This retrospective study aimed to phenotype lung diseases occurring in a well-described pSS-ILD cohort and describe treatment course and outcomes. Between April 2018 and February 2020, all pSS patients attending our Outpatient clinic were screened for possible lung involvement. Clinical, laboratory and high-resolution computed tomography (HRCT) findings were analyzed. Patients were classified according to HRCT findings into five groups: usual interstitial pneumonia (UIP), non-specific interstitial pneumonia (NSIP), desquamative interstitial pneumonia (DIP), combined pulmonary fibrosis and emphysema (CPFE), and non-specific-ILD. Lung involvement was confirmed in 31/268 pSS patients (13%). One-third (10/31) of pSS-ILD patients were Ro/SSA antibody negative. ILD at pSS diagnosis was present in 19/31 (61%) patients. The commonest phenotype was UIP *n* = 13 (43%), followed by NSIP *n* = 9 (29%), DIP *n* = 2 (6 %), CPFE *n* = 2 (6 %), and non-specific-ILD *n* = 5 (16%). Forced vital capacity (FVC) and carbon monoxide diffusion capacity (D_LCO_) appeared lower in UIP and DIP, without reaching a significant difference. Treatment focused universally on intensified immunosuppression, with 13/31 patients (42%) receiving cyclophosphamide. No anti-fibrotic treatments were used. Median follow-up was 38.2 [12.4–119.6] months. Lung involvement in pSS is heterogeneous. Better phenotyping and tailored treatment may improve outcomes and requires further evaluation in larger prospective studies.

## Introduction

Primary Sjögren's Syndrome (pSS) is an increasingly recognized autoimmune disease, primarily affecting secretory gland tissue. Its prevalence is estimated at ~60 cases per 1,00,000 population. The clinical hallmarks are xerophthalmia and xerostomia, however ~30–50% of patients will develop extra-glandular manifestations in a variety of organ systems (1, 2). Lung involvement is relatively common, affecting 9–22% and confers major adverse effects on both life quality and mortality, resulting in a 4-fold increase in 10 years mortality (3, 4).

Lung involvement typically presents with exertional dyspnea and a persisting dry cough. Pulmonary function testing (PFT) is recommended, commonly revealing a reduced carbon monoxide lung diffusion capacity (D_LCO_), and a disproportional loss in forced vital capacity (FVC) (5–7). Abnormalities may be apparent on a standard chest x-ray (CXR), but their absence should not discourage further evaluation for interstitial lung disease (ILD). Pulmonary symptoms can be the first manifestation of pSS. Nannini et al. reported on 105 pSS patients, 10% of whom displayed respiratory manifestations at diagnosis or within the 1st year. At 5 years, prevalence had risen to 20% (+/– 4%) (8). Dry cough was the predominating symptom, affecting 41–61% of patients, with higher than anticipated rates for respiratory infections and pneumonia at 10–35% (3, 9).

Efforts have been made to characterize the relationship between various pSS and interstitial lung diseases, with emphasis upon idiopathic pulmonary fibrosis (IPF). HRCT has been advocated, with a recent systematic evaluation in 527 unselected pSS patients confirming significant interstitial lung changes in 39%. By far the commonest pattern of involvement was non-specific interstitial pneumonia (NSIP), which was observed in 42% of those affected. Usual interstitial pneumonia (UIP), similar in character to IPF, occurred in 11%. Organizing pneumonia (OP) and lymphocytic interstitial pneumonia (LIP) both accounted for <4% of cases. In 82 patients (40%), mixed disease patterns were observed on HRCT (4). The commonest recognized entity is combined pulmonary fibrosis and emphysema (CPFE), which typically consists of upper lobe pan-lobular lung emphysema and basal interstitial features similar to UIP. Usually occurring in smokers, it has also been observed in never smokers (5). LIP has been reported in 10–15% of pSS ILD cases. HRCT imaging reveals thickening of broncho-vascular bundles and interlobular septa, as well as interstitial nodules, ground-glass opacities, and cysts in up to 82% (6, 7, 10).

Within pSS cohorts, ILD has traditionally been linked to smoking, older age, hypergammaglobulinemia, increased rheumatoid factor (RF), or antinuclear antibody titers, anti-SSA or -SSB antibody positivity, elevated C-reactive protein (CRP), and reduced serum C3 levels (10–13). Regarding treatment for pSS ILD, few studies have considered the nature of lung involvement when evaluating efficacy of immunosuppressive drugs, with most being derived from case studies. Corticosteroids together with azathioprine or cyclophosphamide are common in treating pSS-ILD. Many patients, particularly those with UIP, do not appear to benefit from this approach (14–16). Rituximab has been suggested as a universal agent to control pSS-ILD irrespective of form, but data from large studies remains elusive (17).

There is little published information regarding ILD and the typical pSS serological markers and disease activity. No data exists for correlations between biomarkers and the ILD response to immunosuppressive regiments. The primary aim of this study was to systematically evaluate the incidence and characterize ILD phenotype in a well-defined pSS-ILD cohort and summarize outcomes in terms of survival, pulmonary function, serial HRCT scans and response to treatment.

## Methods

### Study Design

A retrospective observational cohort study at a single tertiary care institution was performed.

### Setting

Patients were recruited *a priori* from attendances at both the Rheumatology or Pulmonology outpatient departments of Hannover Medical School between April 2018 and February 2020. Preliminary clinical screening involved identifying patients with new-onset persisting cough and/or exertional dyspnoea New York Heart Association (NYHA) ≥2 associated with any combination of sicca symptoms, myalgia and/or arthralgia or already known patients with pSS and ILD. Patients fulfilling clinical criteria underwent PFT and assessment of various serum markers for autoimmune disease. Based upon these findings, patients suspected of having ILD were referred for HRCT chest imaging in keeping with EULAR Sjögren's syndrome disease activity score (ESSDAI) recommendations (11). Patients fulfilling diagnostic criteria for pSS without pathological lung function and/or imaging formed the control group (Figure S1). All study participants provided written informed consent and the study received Institutional Review Board approval by Hannover Medical School (8179_BO_S_2018).

### Participants

Diagnosis of pulmonary involvement in pSS reflected American College Rheumatology (ACR) and European League Against Rheumatism (EULAR) criteria (12), with a minimum score of 10, which includes shortness of breath or dry cough accompanying abnormal PFT or pathological findings on HRCT scans. pSS classification criteria were applied to all patients reporting either dry eyes or mouth, or those fulfilling at least one positive domain of the ESSDAI with suspected pSS (ESSDAI) (11).

ESSDAI score was calculated for all patients with lung involvement. pSS criteria were met if the combined score for the following items was ≥4: focal lymphocytic sialadenitis and focus score ≥1 (three points) in the labial minor salivary gland biopsy (13), positive anti-SSA (Ro) antibodies (three points), Schirmer test ≤ 5 mm/5 min in at least one eye, or stimulated whole saliva flow rate increase in weight <2.75 g/2 min (one point). In our institute the Saxon test continues to be used to measure xerostomy. It is defined as a stimulated salivary flow test, an increase in weight <2.75 g/2 min is defined as pathological (14). Stimulated and unstimulated salivary flow tests seem to be comparable (15) and the stimulated salivary flow test is still recommended by EULAR in their latest pSS management recommendations (16). Patients presenting with secondary Sjögren's syndrome or possible secondary Sjögren's syndrome with overlap to dermatomyositis or scleroderma were excluded of the study.

Regarding peripheral neuropathy, the same criteria as in a recently published pSS cohort were used (2). All patients underwent Saxon and Schirmer tests, as well as testing for Ro52 and Ro60 antibodies, which were measured quantitatively using EliA by Thermo Fisher (Freiburg, device Phadia250). Patients with one positive test and suspected pSS, underwent a labial minor salivary gland biopsy. Biopsies exhibiting focal lymphocytic sialadenitis with a focus score ≥1, were considered diagnostic of pSS. Patients with biopsies revealing a focus score of <1 did not meet the classification criteria for pSS, and were excluded from the study.

### Variables

Analyses of PFT, HRCT, ESSDAI score, and diagnostic criteria were collated. Furthermore, all treatments for pSS-ILD was documented.

### Data Collection

Non-contrast, HRCT scans were performed using volumetric acquisition, with thin-section reconstruction using maximum 1.5 mm slices, as recommended in the American Thoracic Society/European Respiratory Society (ATS/ERS) guidelines (17). Images were reviewed by a blinded thoracic radiologist, and classified according to Fleischner Society criteria for interstitial lung disease (18, 19).

PFTs were performed on all patients in a dedicated laboratory, consisting of either standard spirometry or body plethysmography in cases with FVC loss suspected of having restrictive ventilatory defects. Diffusion capacity was measured using single-breath determination of carbon monoxide uptake. All tests were performed according to ATS/ERS guidelines (20–22) and results interpreted by blinded pulmonologists. In case of abnormal FVC and or low diffusion capacity (DLCO) chest X-ray (CXR) and in 36 cases HRCT of the chest were performed. Only patients with pathological HRCT scans were included into the analyses.

Follow up PFTs were considered improved if they increased ≥10% of level at treatment initiation, or progressive disease if they decreased ≥10% over treatment baseline. Values remaining between ±10% corridor of baseline were considered stable.

Participants completed a structured questionnaire regarding symptoms, including EULAR Sjögren's syndrome patient reported index (ESSPRI). Furthermore, Saxon and Schirmer tests, salivatory gland biopsy, if necessary, as well as diagnostic work up for ESSDAI scoring were performed (11). In keeping with routine departmental protocols, all clinical and diagnostic data were prospectively archived in a customized Microsoft® Access database (Microsoft Corporation, Redmond, WA, USA).

### Statistical Analysis

Descriptive statistics were calculated using R version 3.6.0 (Foundation for Statistical Computing, Vienna, Austria) in conjunction with “Hmisc” (Frank E. Harrell Jr. (2020). Hmisc: Harrell Miscellaneous. R package version 4.3-1.), “dplyr” (Hadley Wickeam, Romain Fracois, Lionel Henry, and Kirill Müller (2020). Dplyr: A Grammar of Data Manipulation. R package version 0.8.5.) and “ggplot2” (H. Wickam. ggplot2: Elegant graphics for data analysis. Springer-Verlag New York, 2016) packages. To compare age of pSS onset between ILD and non-ILD controls, a non-parametric age distribution was assumed and a two-sided Wilcoxon signed-rank test was used, *p* < 0.05 were considered statistically significant. Values reported are median [inter-quartile range] unless otherwise reported.

## Results

**Figure 1:**
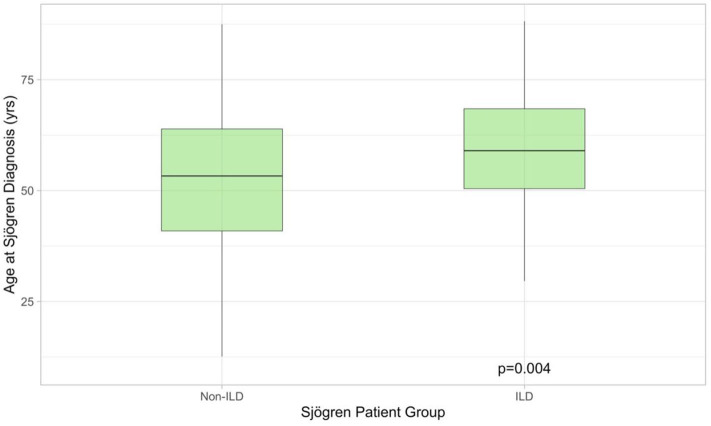
Boxplot illustrating age at which Sjögren's syndrome was diagnosed in patients with (*n* = 31) and without (*n* = 268) lung involvement. The former were older at presentation. ILD, interstitial lung disease.

Two-hundred and sixty-eight patients with pSS were identified, of whom 51/268 had clinical symptoms like dry cough or shortness of breath. All symptomatic patients underwent PFT, 36/51 had pathological findings defined as FVC ≤ 80% predicted and/or DLCO ≤ 70% predicted. HRCTs were performed on all 36 patients. Of these, 31/36 (86%) exhibited pathological findings possibly related to pSS. Five patients had no changes suggestive of ILD and were excluded from the analysis. In total 31/268 (13%) pSS patients had ILD. Demographics for pSS-ILD patients are summarized in Table 1. The majority of patients were never-smoking females, presenting in their seventh decade. All were Caucasian. Compared to non-ILD patients (*n* = 237), pSS-ILD patients were significantly older (59.0 [50.4–68.5] vs. 53.3 [40.9–63.9] years; Wilcoxon Rank Sum *p* = 0.0044, Figure 1) at time of pSS diagnoses. Median follow-up was 38.2 [12.4–119.6] months.

**Table 1:** Summary of patient demographics.

**Cohort demographics (*****n*** **=** **31)**		
Female, *n* (%)	22	(71)
Never smoking, *n* (%)	23	(74)
Initial manifestation (ILD), *n* (%)	17	(71)
Age 1st manifestation, years	58.9	[49.6–68.4]
– ILD as 1st manifestation, *n* (%)	19	(61)
– Time to pSS diagnosis in ILD first, months	6.2	[3.1–44.1]
– Time to ILD diagnosis in pSS first, months	3.1	[0.0–38.8]
Follow up, months	38.2	[12.4–119.6]
**EULAR Sjögren's Syndrome Disease Activity Index (ESSDAI)**		
Constitutional Symptoms, *n* (%)	1	(3)
Lymphadenopathy, *n* (%)	1	(3)
Glandular involvement, *n* (%)	2	(7)
Articular involvement, *n* (%)	12	(39)
Cutaneous involvement, *n* (%)	3	(10)
Pulmonary involvement, *n* (%)	31	(100)
Renal involvement, *n* (%)	1	(3)
Muscular involvement, *n* (%)	1	(3)
Peripheral nervous system involvement, *n* (%)	6	(19)
Central nervous system involvement, *n* (%)	1	(3)
Hematological involvement, *n* (%)	15	(48)
Biological involvement, *n* (%)	9	(29)
**Laboratory values at ILD diagnosis**		
CRP >10 mg/l, *n* (%)	13	(42)
Rheumatoid factor positive, *n* (%)	16	(52)
ANA >1:160, *n* (%)	28	(90)
Presence of SSA (Ro) antibody, *n* (%)	21	(68)
Presence of SSB (La) antibody, *n* (%)	7	(23)
xANCA positive, *n* (%)	3	(10)
**Xerostomia tests**		
Saxon test pathological, *n* (%)	12^a^	(41)
Schirmer test pathological, *n* (%)	27^a^	(87)
**Salivary gland biopsy**		
Chisholm Mason- grade ≥3, *n* (%)	11^b^	(85)
**Lung function at ILD diagnosis**		
% Predicted forced vital capacity (FVC)	65	[52–88]
% Predicted diffusing capacity (D_LCO_)	48	[41–80]
**CT patterns of lung disease**		
Usual interstitial pneumonia, *n* (%)	13	(42)
Non-specific interstitial pneumonia, *n* (%)	9	(29)
Desquamative interstitial pneumonia, *n* (%)	2	(7)
Combined pulmonary fibrosis and emphysema, *n* (%)	2	(7)
Unspecific interstitial change, *n* (%)	5	(16)
**Treatment**		
**1st line DMARD**		
– *Cyclophosphamide, n (%)*	13	(42)
– *Azathioprine, n (%)*	8	(26)
– *Methotrexate, n (%)*	5	(16)
– *Hydroxychloroquine, n (%)*	2	(7)
– *Rituximab, n (%)*	1	(3)
– *Mycophenolate mofetil, n (%)*	1	(3)
Number of treatment modalities attempted per patient	3	[2–3]

*Clinical, laboratory, pulmonary function, and computer tomography findings at the time of original diagnosis have been included*.

a *Results available for 29/31 patients included in the cohort*.

b *Results available for 13/31 patients included in the cohort*.

*Values represent median [inter-quartile range] unless otherwise stated*.

*ILD, interstitial lung disease; pSS, primary Sjögren's Syndrome; CRP, C reactive protein; ANA, antinuclear antibody; xANCA, atypical anti-neutrophil cytoplasmatic antibodies; DMARD, disease modifying anti-rheumatic drug*.

At ILD diagnosis, the median ESSDAI was 19.0 [14.3–24.8]. After lung involvement, the most common domains of disease activity were hematological (15/31, 48%), joints (12/31, 39%), and biological (9/31, 29%). The latter derived from hypergammaglobulinemia and or hypocomplementemia or presence of cryoglobulinemia. In nine patients (29%), both the Saxon and Schirmer tests were pathological, with one or other being positive in 22/31 patients (71%). Of the 10 SSA (Ro)-antibody negative patients (31%), whilst half were also Saxon test negative all had salivary gland biopsies ≥ Chisholm Mason grade 3. Four patients without objective xeropthalmia and xerostomia were diagnosed with pSS due to positive anti-SSA(Ro) antibodies and focal sialadenitis.

At ILD diagnosis PFTs demonstrated impaired ventilation and diffusion in almost all patients, with median forced vital capacity (FVC) being 65 [52–88]% predicted and median DLCO of 48 [41–80]% predicted. Patients demonstrating UIP and in particular desquamative interstitial pneumonia (DIP) were most severely affected with FVC of 60 [−46-65]% predicted and 50 [45–55]% predicted at diagnosis, along with DLCO of 53 [36–71]% predicted, and 35 [29–41]% predicted respectively (Table 2).

**Table 2:** Subgroup demographics with respect to interstitial lung disease patterns, as determined in the original CT Thorax.

	**UIP**		**NSIP**		**DIP**		**CPFE**		**Unspecific**	
Patient, *n* (%)	13	(43)	9	(29)	2	(6)	2	(6)	5	(16)
Female, *n* (%)	9	(69)	5	(56)	2	(100)	1	(50)	3	(60)
Age Onset, years	58.9	[50.6–68.8]	57.3	[50.0–64.3]	36.8	[36.2–37.4]	60.6	[43.5–77.8]	67.8	[63.9–68.3]
Age SS, years	59.0	[50.7–68.8]	57.7	[50.2–68.4]	45.7	[37.5–53.9]	60.7	[43.6–77.9]	67.9	[64.0–68.4]
Age ILD, years	61.9	[54.9–69.3]	57.4	[51.1–64.4]	36.9	[36.2–37.5]	62.7	[47.6–77.9]	68.4	[65.9–70.8]
Never smoker, *n* (%)	10	(77)	6	(67)	1	(50)	1	(50)	5	(100)
– Pack years	2		9		3		18		-	
ESSDAI score	18	[14–25]	17	[15–22]	19	[13–25]	12	[10–14]	22	[21–25]
**Lung function at ILD diagnosis**										
FVC, % pred	60	[46–65]	70	[54–76]	50	[45–55]	98	[93–102]	79	[78–98]
D_LCO_, % pred	53	[36–71]	47	[41–68]	35	[29–41]	70	[55–84]	79	[63–84]
**Treatment**										
**1st line treatment**										
*CYC, n (%)*	6	(45)	5	(56)	2	(100)	0	-	0	-
*AZA, n (%)*	4	(31)	1	(11)	0	-	1	(50)	2	(40)
*MTX, n (%)*	1	(8)	1	(11)	0	-	1	(50)	2	(40)
*HCQ, n (%)*	0	-	1	(11)	0	-	0	-	1	(20)
*RTX, n (%)*	1	(8)	0	-	0	-	0	-	0	-
*MMF, n (%)*	1	(8)	0	-	0	-	0	-	0	-
Number treatments	3	[2–4]	3	[2–3]	2	[1–3]	3	[1–4]	2	[2–3]
**Treatment outcomes**										
**FVC**										
Improved, *n* (%)	2	(15)	1	(12)	2	(100)	0	-	0	-
Stabilized, *n* (%)	8	(62)	4	(44)	0	-	2	(100)	3	(100)
Declined, *N* (%)	3	(23)	4	(44)	0	-	0	-	0	-
**D**_**LCO**_										
Improved, *n* (%)	3	(23)	1	(11)	2	(100)	0	-	1	(20)
Stabilized, *n* (%)	9	(69)	5	(56)	0	-	2	(100)	4	(80)
Declined, *N* (%)	1	(8)	3	(33)	0	-	0	-	0	-
Follow up, months	37	[12–96]	17	[13–50]	113	[9–218]	65	[10–120]	170	[81–182]
Deaths, *n* (%)	1	(8)	0		0		0		0	

*Lung function outcomes based upon ±1% per month change over baseline values at original diagnosis*.

*UIP, usual interstitial pneumonia; NSIP, non-specific interstitial pneumonia; DIP, desquamative interstitial pneumonia; CPFE, combined pulmonary fibrosis and emphysema; SS, Sjögren's Syndrome; ILD, interstitial lung disease; ESSDAI, EULAR Sjögren's Syndrome Disease Activity Index; FVC, forced vital capacity; D_LCO_, Carbon monoxide lung diffusion capacity; CYC, cyclophosphamide; AZA, azathioprine; MTX, methotrexate; HCQ, hydroxychloroquine; RTX, rituximab; MMF, mycophenolate mofetil*.

*Values represent median [inter-quartile range] unless otherwise stated*.

Analysis of the CT imaging revealed that UIP was the predominating pattern of disease (*n* = 13, 42%), with NSIP also proving common (*n* = 9, 29%). Similar numbers of the much more aggressive DIP and multi-factorial CPFE were observed (both *n* = 2, 6%).

**Figure 2:**
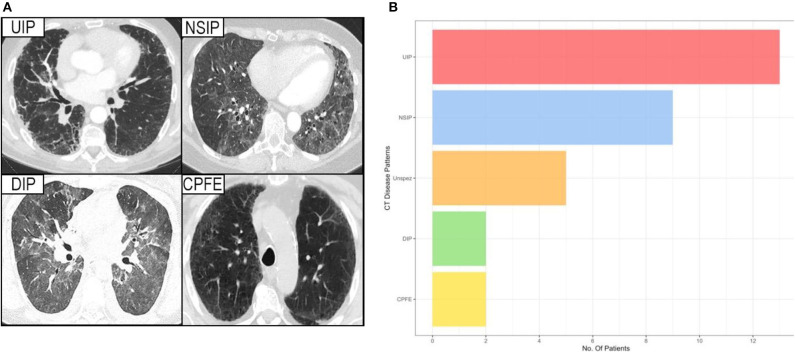
**(A)** Showing the HRCT pattern of the various interstitial lung diseases. **(B)** Showing the prevalence of different forms of interstitial lung disease among patients with a proven Sjögren's syndrome, considered contributory to their lung disease. UIP, usual interstitial pneumonia; NSIP, non-specific interstitial pneumonia; DIP, desquamative interstitial pneumonia; CPFE, combined pulmonary fibrosis and emphysema; Unspez, unspecific interstitial changes.

The remaining five patients exhibited various different patterns of lung involvement including bronchiectasis, tree in bud phenomena suggestive of bronchiolitis, and in one patient cystic changes suggestive of LIP. Due to the small numbers these cases were amalgamated into non-specific disease for the purposes of analysis (Figure 2).

**Figure 3:**
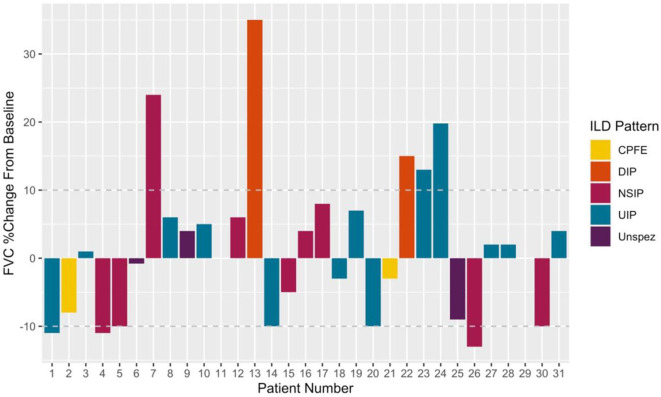
Illustrating %changes in FVC following treatment initiation. Deltas calculated using last available measurement. Disease progression (treatment failure) was defined as a 10% fall in FVC from that recorded at treatment initiation. A 10% improvement symbolized Improved on treatment. Patients within ± 10% baseline were classed as stable.

Subgroup analysis revealed a persisting female predominance across all forms of lung involvement. Patients exhibiting a DIP pattern presented much earlier. Lung function revealed more profound ventilatory impairment in UIP and DIP compared to other phenotypes. A similar, albeit less obvious, pattern was observed in diffusion coefficients. Regarding 1st line treatment, no clear patterns reflecting pulmonary phenotype were identified and again the small numbers prevented statistical appraisal. During the 29.0 [8.9–80.5] months of follow-up after ILD diagnosis, 24/31 patients achieved stabilized or improved FVC after commencing treatment (Figure 3). One patient died during follow-up due to an unrelated cancer. It should be noted however, that follow-up in the predominant pulmonary phenotypes remains limited (UIP 18.0 [9.5–97.8] months; NSIP 12.0 [8.9–49.7] months).

In general terms, 1st line treatment consisted of systemic corticosteroids in conjunction with disease modifying anti-rheumatic drugs (DMARDs). Cyclophosphamide was most commonly used (*n* = 13, 42%), followed by azathioprine and methotrexate (*n* = 8, 26% and *n* = 5, 16%). In 26/31 patients (84%) the 1st line DMARD was changed. The median numbers of different DMARDs used was 3 [2–3], with the maximum used in an individual patient being seven.

Evaluating 1st line treatment choice, patients commenced on cyclophosphamide tended to be younger at median 58.4 [47.6–67.2] years and have poorer lung function (median FVC 64 [44–88]% predicted) compared to the other DMARDs used. Given the limited data available no statistical analysis has been performed.

In terms of initial treatment response, across all groups and independent of treatment received, a gradual slowing in FVC loss was observed, with a suggestion of some recovery among those patients with the longest follow-up. These trends were more apparent in the larger NSIP and UIP subgroups but the limited follow-up prevents meaningful interpretation of these preliminary findings (Figure S2). Follow-up HRCT Thorax has to date been performed in 13/31 patients (42%) at a median 7.0 [4.1–17.8] months after the first scans. A small minority of patients (6/13, 46%) demonstrated radiological progression. This included both DIP patients who had received cyclophosphamide, as well as 2/9 (22%) NSIP patients who received hydroxychloroquine or cyclophosphamide respectively, a CPFE patient (1/2, 50%) who received methotrexate and a single UIP patient (1/13, 8%) who initially received mycophenolate. Nonetheless none of the patients have died due to their lung disease or required lung transplantation during ongoing follow-up, or have been commenced on additional anti-fibrotic therapies, such as pirfenidone or nintedanib.

## Discussion

Our data raises a number of important aspects regarding ILD and pSS despite small cohort size and limited follow-up. Firstly, symptomatic lung involvement was identified in 13% of pSS patients attending our institution. This corroborates previously reported prevalence ranging from 9–22% (23, 24) and supports initiating structured screening for lung disease in pSS patients. Normal lung physiology features inherent functional reserves and inevitably significant pathology exists before patients become symptomatic. This is common in many lung diseases, partially accounting for disappointing outcomes in chronic respiratory conditions including ILD.

The second important implication is the need for effective pSS screening in patients presenting with apparently idiopathic ILD. Reliance upon antibody testing for anti-SSA (Ro) and anti-SSB (La) appears inadequate for screening, with our results suggesting that up to one-third of patients could be missed. Potential advantages of augmenting screening with testing for dry eyes and mouth and in equivocal cases proceeding to salivary gland biopsy requires further careful evaluation.

Rheumatologists regularly evaluate ILD patients on respiratory wards or as outpatient pulmonology referrals. Interdisciplinary cooperation may explain the high percentage of ILD patients, in whom pSS was subsequently diagnosed on routine screening (9/31, 29%) compared to previous reports. of 10% (8). This raises the possibility that significant numbers of ILD patients with undetected pSS may be missed or managed as IPF instead. Recently, interstitial pneumonia with autoimmune features (IPAF) was defined by the American Thoracic Society (ATS)/European Respiratory Society (ERS) as an interstitial pneumonia with clinical, serological, or morphological features of an autoimmune disease without fulfilling criteria for a specific connective tissue disease (CTD) (25). In such cases, we would advocate additional diagnostic work up for pSS-ILD given the heterogeneity of the latter, which lies well within the IPAF criteria.

Compounding this further, is the diversity of ILD observed in the cohort. A persisting misconception remains, that NSIP is the predominating HRCT phenotype occurring in autoimmune connective tissue diseases (4, 26). Our data could not corroborate this, with UIP actually being the most common manifestation. Unquestionably, the data presented is circumstantial and no causality can be inferred. It remains possible that our results merely reflect different conditions occurring in the same patient. Contradicting this however is the predominance of never-smoking females with UIP. Nevertheless, the data reiterates the need for critical appraisal of ILD phenotypes as a catalyst for further research and potentially, individualized treatment. Current data for UIP in pSS is limited, with case reports suggesting a poor response to augmented immunosuppression (1). Although our data does not support these results, it should be reiterated that our experiences are greatly limited, in terms of both numbers and duration of follow-up. It should be noted however that reports suggesting more favorable outcomes in CTD-associated UIP compared to idiopathic UIP (27).

Contradictory data exists regarding the age of diagnosis in pSS with ILD and without. Whilst Dong et al. report a significant age difference 57.44 (+/– 14.08) years in pSS patients without ILD vs. 61.00 (+/– 11.23) years in patients with ILD, Palm et al. did not see a difference (4, 23, 28). In our cohort pSS patients with ILD were older than patients without ILD at time of diagnosis.

The choice of treatment in our cohort was based on severity of lung involvement and HRCT findings. If alveolitis was detected and severe impairment of lung function was present, glucocorticoids in combination with intravenous cyclophosphamide was used as first line treatment. If possible six courses of cyclophosphamide (15 mg/kg) were given monthly followed by a maintenance therapy of mycophenolate mofetil or azathioprine.

In January 2020 EULAR recommendations for the management of pSS have been published, as first line treatment for moderate and high ESSDAI score glucocorticoids 0.5–1 mg/kg are recommended regarding to severity. As second line immunosuppressive agents are suggested, and as a rescue therapy cyclophosphamide and rituximab are recommended. But the task force points out that there are no controlled studies or head to head comparisons of any immunosuppressive agents allowing support of a differentiated organ-guided therapeutic approach (16). Which highlights the necessity of therapy studies in pSS patients with extra-glandular manifestations.

Research into UIP treatments in non-pSS populations has received a great deal of attention in the past decade. Traditional treatments with steroids, azathioprine, and n-acetyl-cysteine, based on IFIGENIA study (29) were called into question by the extended PANTHER-IPF study (30) which suggested that immunosuppression was actually worsening prognosis in UIP-ILD. This combined with the early results from the CAPACITY (31) and ASCEND (32) trials led to a paradigm shift away from immunosuppression and toward novel anti-fibrotic agents. Beyond idiopathic pulmonary fibrosis, recent data from the SENSCIS study (33) examined the effects of nintedanib in systemic sclerosis associated lung disease. This prospective, randomized, placebo controlled study included over 570 patients and demonstrated clinical benefit. Current publications from this cohort have not yet attempted to describe or phenotype ILD in these patients.

Our cohort included only one patient with HRCT features suggestive of LIP, which may just reflect the small size of our cohort. Furthermore, LIP is a histological diagnosis, biopsies were performed only in 4/31 patients, so that no meaningful statistical analyses was possible.

Nevertheless, our cumulative findings reinforce the need for continued refinement of disease phenotypes and evaluation of tailored treatment approaches. Due to its limitations, the data presented here is at best preliminary and serves principally as a basis for focusing future research. Our cohort is small, the data collection was entirely retrospective and both evaluation**—**in terms of lung function and HRCT scanning**—**contains inevitable selection bias. In certain populations, HRCT in asymptomatic patients have confirmed pathological interstitial findings (4). In our institution performance of HRCT in asymptomatic patients is ethically difficult. To compensate for this, the PFT criteria are intended to allow very early detection, to minimize the number of potential missed cases. Crucially, no reliable screening has been performed in asymptomatic patients. Compounding this further, was the reliance on lung function and HRCT imaging rather than histological confirmation. Regarding PFT, analysis was based on FVC values, rather than lung volumes such as total lung capacity (TLC). FVC has been almost universally employed in large multi-center IPF studies due to logistical concerns. TLC measurements on body plethysmography offer clear advantages in identifying and monitoring ILD, but is both time consuming and expensive. Similar issues exist with transbronchial and open-lung biopsies, notwithstanding the additional patient risk such procedures entail.

In conclusion, our results reveal that pulmonary disease is commonly associated with pSS, manifesting in a variety of different clinical entities. Screening for pSS in patients with unclear lung disease should be performed regardless of subjective sicca symptoms via screening for xeropththalmy or xerostomy and in case of unremarkable antibodies a salivary gland lip biopsy should be performed. Based upon existing data from other disease groups, potential exists for improving outcomes by refining disease recognition strategies and designing appropriate studies with aim of structured surveillance and tailored treatment strategies.

## Data Availability Statement

The datasets generated for this study are available on request to the corresponding author.

## Ethics Statement

The studies involving human participants were reviewed and approved by ethics committee of Hannover Medical Highschool. The patients/participants provided their written informed consent to participate in this study.

## Author Contributions

DE, AJ, GS, and SH: conception and design of the study, acquisition and interpretation of data, drafting the article, and final approval of the version to be submitted. TSk, TW, and RS: interpretation of data, revising critically for important intellectual content, and final approval of the version to be submitted. JH: acquisition of data, analyzing all CT reports, revising critically for important intellectual content, and final approval of the version to be submitted. TT and TSe: acquisition and interpretation of data and final approval of the version to be submitted. All authors take responsibility for all aspects of the reliability and freedom from bias of the data presented and their discussed interpretation.

## Conflict of Interest

The authors declare that the research was conducted in the absence of any commercial or financial relationships that could be construed as a potential conflict of interest.
