# Supplementary material for: Lung Involvement in Primary Sjögren's Syndrome—An Under-Diagnosed Entity
Source: Front Med (Lausanne). 2020 Jul 16;7:332. doi: 10.3389/fmed.2020.00332 (PMC7378373; doi:10.3389/fmed.2020.00332)
Supplement: Supplementary file 1 [file Image_1.pdf]

# Lung Involvement In Primary Sjögren Syndrome – An Under-Diagnosed Entity

## Supplementary data Fig.S1

Fig. S1: Flow Chart showing the selection of patients with primary Sjögren Syndrom (pSS) and lung involvement.

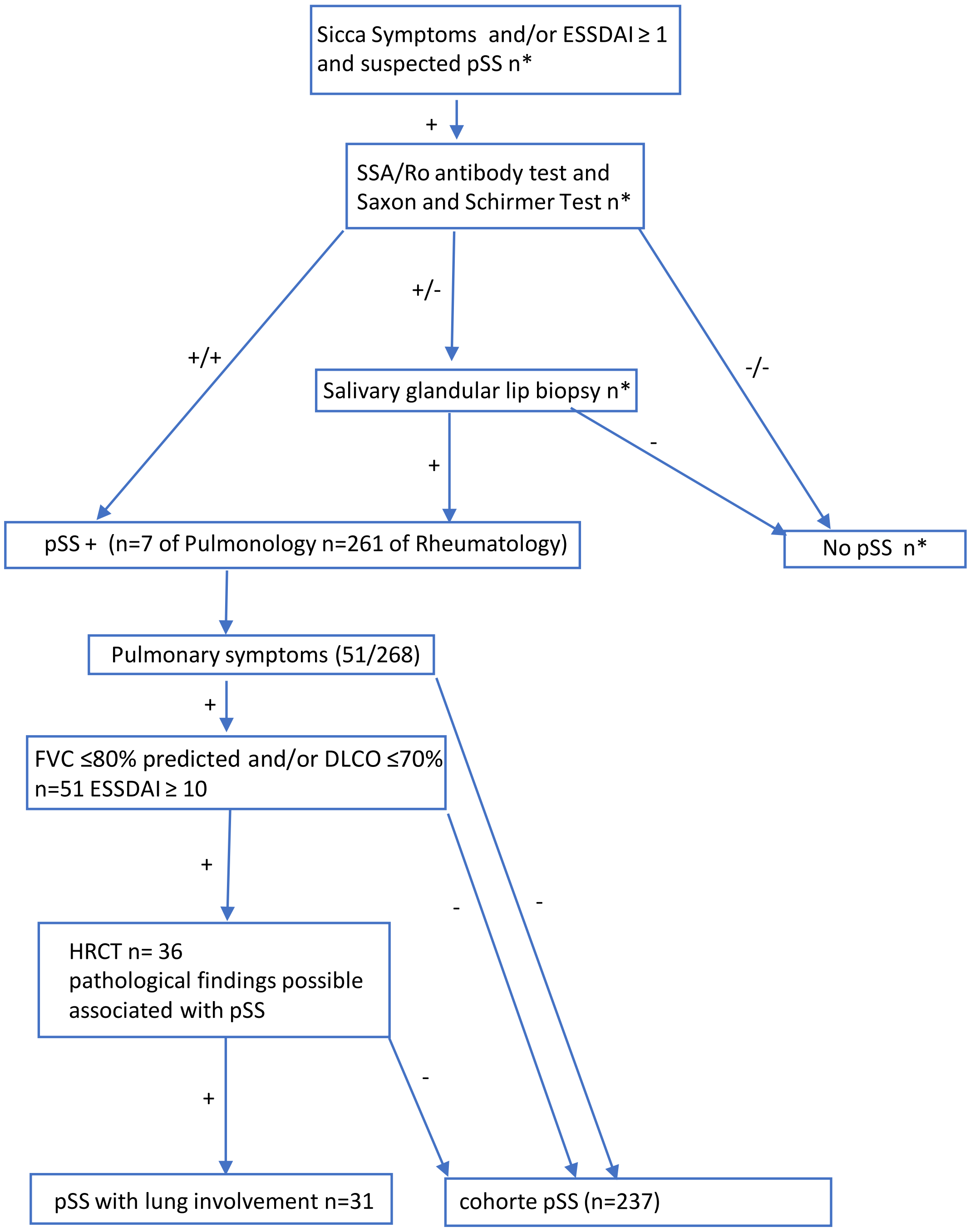


N* numbers not documented, HRCT High Resolution Computed Tomography, ESSDAI EULAR Sjögren´s syndrome disease activity score, FVC forced vital capacity, DLCO diffusion capacity
