# Supplementary material for: Lung Involvement in Primary Sjögren's Syndrome—An Under-Diagnosed Entity
Source: Front Med (Lausanne). 2020 Jul 16;7:332. doi: 10.3389/fmed.2020.00332 (PMC7378373; doi:10.3389/fmed.2020.00332)
Supplement: Supplementary file 2 [file Image_2.pdf]

Lung Involvement In Primary Sjögren Syndrome – An Under-Diagnosed Entity

Supplementary data Fig.S2

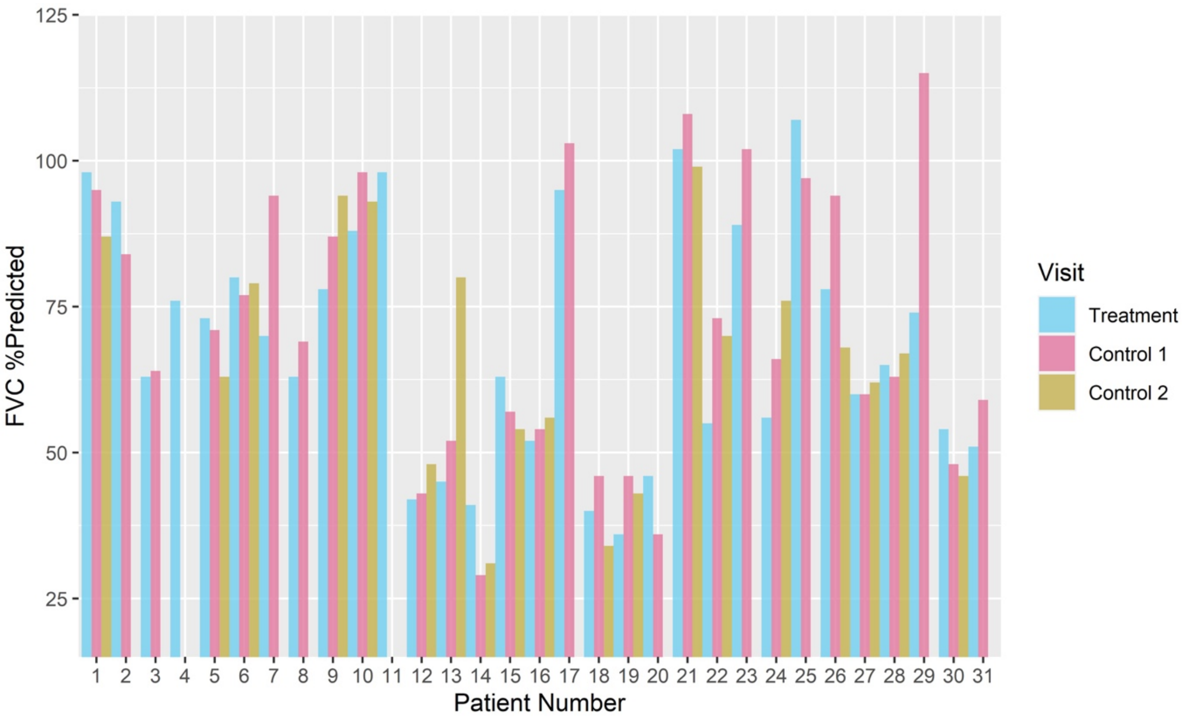

Fig.S2 Showing follow up FVC at time of treatment initiation and subsequent first and second lung function controls. Mean time to first control was 9 and second control 17 months.
FVC: Forced Vital Capacity
